# Effect of Mobile Carrier on the Performance of PVAm–Nanocellulose Facilitated Transport Membranes for CO_2_ Capture

**DOI:** 10.3390/membranes11060442

**Published:** 2021-06-12

**Authors:** Riccardo Casadei, Elham Firouznia, Marco Giacinti Baschetti

**Affiliations:** Dipartimento di Ingegneria Civile, Chimica, Ambientale e dei Materiali (DICAM), Alma Mater Studiorum—Università di Bologna, Via Terracini, 28, 40131 Bologna, Italy; riccardo.casadei11@unibo.it (R.C.); elham.firouznia@studio.unibo.it (E.F.)

**Keywords:** nanocellulose, polyvinylamine, L-arginine, facilitated transport, CO_2_ capture

## Abstract

Facilitated transport membranes obtained by coupling polyvinylamine with highly charged carboxymethylated nanocellulose fibers were studied considering both water sorption and gas permeation experiments. In particular, the effect of the L-arginine as a mobile carrier was investigated to understand possible improvements in CO_2_ transport across the membranes. The results show that L-arginine addition decreases the water uptake of the membrane, due to the lower polyvinylamine content, but was able to improve the CO_2_ transport. Tests carried on at 35 °C and high relative humidity indeed showed an increase of both CO_2_ permeability and selectivity with respect to nitrogen and methane. In particular, the CO_2_ permeability increased from 160 to about 340 Barrer when arginine loading was increased from 0 to 45 wt%. In the same conditions, selectivity with respect to nitrogen was more than doubled, increasing from 20 to 45. Minor improvements were instead obtained with respect to methane; CO_2_/CH_4_ selectivity, indeed, even in presence of the mobile carrier, was limited to about 20.

## 1. Introduction

The increased awareness of issues related to global warming has in recent years generated a strong push towards the reduction of greenhouse gas emissions in the atmosphere [1,2]. Among the different strategies needed to reach the reduction goals set by The Paris Agreement [3], carbon capture, focusing on separation of CO_2_ from effluent streams before their dispersion in atmosphere, still plays an important role [4,5] and has driven a number of studies towards the improvement of CO_2_ capture technologies to make this separation cheaper and more efficient [6,7,8].

Membrane separation is one of the techniques that has been considered for such an application [9,10], and facilitated transport membranes (FTMs) have often been considered suitable for the field of post-combustion carbon capture, due to the ability to obtain high selectivity and permeability at low pressure [11].

FTMs indeed rely on the interaction of the target molecules with fixed or mobile carriers, which are able to transport them from one side of the membrane to the other, thus creating an additional transport mechanism, which can overcome most of the limitations of solution diffusion membranes [12,13,14].

For acidic gases such as carbon dioxide, the considered carriers are often based on alkaline species such as salts, aminated compounds and amino acids [15,16,17,18,19]. Aminated polymers in particular have been used as base materials for a number of membranes due to the possibility of transporting CO_2_ through different pathways, related to the formation of bicarbonate (II) or carbamate ions (I) [14,20,21]:

(I) CO_2_ + H_2_O + R–NH_2_ ⇄ HCO_3_^−^ + R–NH_3_^+^
(II) 2 R–NH_2_ + CO_2_ ⇄ R–NH–COO^−^ + R–NH_3_^+^

In both pathways, water is of extremely high importance as it participates in the reactions and generally increases the mobility of the ionic species in the materials. For this reason, facilitated transport membranes usually have high efficiency in highly humid conditions. This fact poses problems in terms of resistance of aminated polymers, which are usually water soluble and lose their mechanical stability at RH conditions, which allows the facilitated transport to become fully efficient. For this reason, they are usually coupled with other polymers that serve as mechanical support. Poly-amine membranes, therefore, are often considered in composition with other polymers such as polyvinyl alcohol (PVOH) and crosslinking agents [16,22,23,24]. The decrease in permeability usually caused by the crosslinking is then counterbalanced by the addition of different mobile carriers.

In recent years, another approach has also been considered that uses nanofillers in order to give the membrane the needed strength and increase permselective efficiency. Graphene- and silica-based nanofillers [25,26,27] as well as carbon nanotubes [28,29,30] and metal–organic frameworks (MOFs) [31] were considered for this purpose along with, more recently, nanocellulose in forms of fibers or nanocrystals [19,32,33,34,35,36,37,38].

Due to its high mechanical properties [39,40] and strong hydrophilic characteristics [41,42], nanocellulose offers a perfect match for aminated polymers in order to obtain mechanically strong hydrophilic membranes. Ansaloni et al. [33] showed that nanocellulose in humid conditions can have CO_2_ selectivity above 600 with respect to nitrogen but low permeability (~10 Barrer^1^). The permeability, however, can be increased by mixing the material with commercial polyvinylamine (PVAm). Janakiram et al. [36] obtained interesting results by loading PVOH /polyallylamine blends with different surface-modified nanocellulose. Permeance of up to 650 GPU^1^ and a separation factor of up to 50 were obtained in CO_2_/N_2_ mix gas tests for the system where nanocellulose modified with four-arm branched polyethyleneglycol molecules was adeed. Venturi et al. [19] obtained good results by considering carboxymethylated nanofibrillated cellulose (cNFC) mixed with arginine, which acted as a mobile carrier. In particular, CO_2_ permeability values in the order of 220 Barrer were obtained, which, coupled with a CO_2_/N_2_ selectivity above 180, allowed the membrane to surpass Robeson’s upper bound [43].

Arginine, which is often considered the most hydrophilic among the amino acids [44], is indeed known to have high affinity for CO_2_ and has been used not only in membranes but also in absorption [45,46] and adsorption systems [46,47] focused on CO_2_ separation. In the case of membranes, very interesting results were obtained by coupling it with chitosan [18]; the material obtained indeed showed permeability higher than 1000 Barrer at high temperatures and selectivity well above 100. Other examples were related to its use in conjunction with PVOH hollow fibers [48], which showed a 40% increase of CO_2_ permeance upon addition of 30 wt% amino acid, or in sulfonated poly(ether ketone) (SPEEK), which reached Robeson’s 2008 upper bound upon addition of 20% arginine salts CO_2_ [49].

In view of these results and in order to further extend the analysis and the optimization of nanocellulose-based membranes for carbon capture applications, in the present work, ternary mixtures of PVAm/cNFC and arginine were tested to explore their potential in view of CO_2_ separation from nitrogen and methane. Permeation experiments carried out at 35 °C and high RH were coupled with water sorption tests in order to gain further insight into the role of water in the membrane performance.

## 2. Materials and Methods

### 2.1. Materials

**Figure 1:**
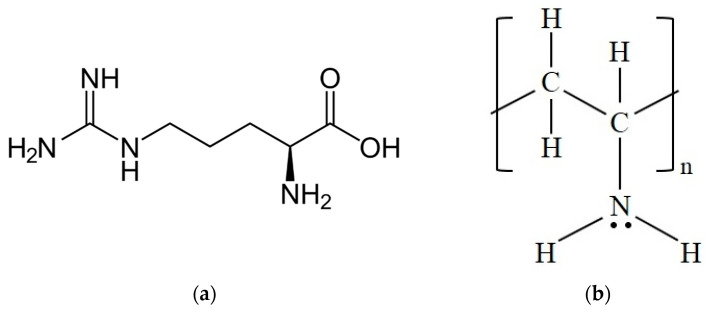
Chemical structure of (**a**) L-arginine and (**b**) (neutral) PVAm.

In the present work, polyvinylamine and L-arginine, the structures of which are shown in Figure 1, were considered as fixed and mobile CO_2_ carriers, respectively, within the membrane. Nanocellulose was added in order to increase membrane stability and mechanical properties in humid conditions.

PVAm is a weak linear cation polyelectrolyte: the amine group’s protonation is possible in acidic conditions, and the charge density as well as the polymer structure in solution are a function of the pH [50,51]. Due to its extremely high primary amine content, which can trigger the facilitated transport of CO_2_, it has been widely studied as a matrix for FTMs [22,25,29,30,31,32,33].

The PVAm used in the present work was Lupamin^®^9095, a commercial form of PVAm kindly provided by the producer (BASF Italia s.p.a, Cesano Maderno, Italy). Lupamin^®^9095 consists of an aqueous solution of a copolymer made of polyvinylamine and polyvinylformamide at high molecular weight (≈340,000 Da), with a hydrolysis degree higher than 90%, which represents the number of amine groups relative to formamide groups. Solid content is about 20–22 wt%, which includes the copolymer and sodium formate salts, which are byproducts of production process [25].

The high amount of salt caused aggregates in the dry membranes, reducing their homogeneity, and made them prone to excessive swelling in presence of water; therefore, it was removed prior to membrane preparation. The purification protocol, described in a previous work [25], could reduce the presence of salts up to 70% with respect to the amount initially present in the commercial product.

Arginine is a polar chiral amino acid, and its L-enantiomer represent one of the essential amino acids in humans. As with many other amino acids, arginine has attracted interest as a mobile carrier in facilitated transport membranes due to the high amine content, which allows good interactions with CO_2_ [18,19,45,46,47,48,49,50,52]. Furthermore, relative to other amino acids, arginine is cheap and has a high molar mass (174 g/mol) and high decomposition temperature (about 230 °C), characteristics that may help in preventing leakage from membranes, which is one of the main concerns when using mobile carriers. L-arginine powder used in the present work was purchased from Sharlab Italia s.r.l. (Riozzo, Italy) and used without any further processing.

Nanocellulose used for membrane preparation was kindly provided by INOFIB (Saint-Martin-d’Hères Cedex, France) and was synthesized from eucalyptus cellulose fibers, which were carboxymethylated and then mechanically treated with a Matsuko Grinder. The overall production protocol was based on the method developed by Wågberg et al. [53] and has been reported elsewhere [19]. The result was a 2.9 wt% suspension of carboxymethylated-nanofibrillated cellulose (cNFC) with nanofibrils with diameter in the order of 80 to 150 nm and a surface charge of about 3600 µequiv/mol as reported by the producer.

### 2.2. Membrane Preparation

Three different types of membrane were considered in the present work, one obtained by mixing cNFC with an equal amount of PVAm and two obtained by adding different amounts of arginine to the previous solution; the overall concentrations are reported in Table 1.

The membrane preparation procedure had to be modified with respect to previously reported protocols [32,33] as the use of purified PVAm and nanocellulose with high surface charge led to immediate precipitation of white aggregates upon mixing [54]. For this reason, the suspension used for membrane casting was obtained by slowly adding the PVAm (2.5 wt% aqueous solution) in a dilute cNFC suspension vigorously mixed (5000 rpm) with a Ika Ultra Turrax T18 Digital homogenizer (IKA^®^-Werke GmbH, Staufen, Germany). After homogenization, the suspension was sonicated for at least 3 min in order to remove gas bubbles and stabilize the mixtures and was then poured in a glass Petri dish, covered with perforated aluminum foil and placed in a ventilated oven at 35 °C until complete water evaporation.

The same procedure was used for the L-arginine containing films in this case; however, the amino-acid powder was added to the cNFC suspension before PVAm to reduce the tendency of aminated polymer to interact with nanocellulose causing precipitation. Ternary solution indeed was more stable the binary one with less tendency to precipitate. As far as the preparation procedure is concerned, the cNFC/arginine suspension was obtained directly by pouring arginine powder into the nanocellulose suspension, which was then stirred for an hour at 500 rpm to completely dissolve the amino acid before starting the PVAm addition protocol.

The films obtained were homogenous and did not shown any sign of agglomeration. Their thickness was measured through a disc micrometer (Mitutoyo, Series 227-221, Mitutoyo Europe GmbH, Neuss, Germany) and was in the order of 50 to 60 µm with variations in each sample, which were always lower than 10%.

### 2.3. Fourier Transform Infrared Spectroscopy (FTIR)

Different membranes produced in the present work were analyzed through Fourier transform infrared spectroscopy with attenuated total reflection setup (FTIR-ATR), which can be used to characterize solid materials (the membrane samples, in this case) through the analysis of the IR absorption in an evanescent wave that penetrates into the sample through the ATR crystal beneath it, thus simplifying the sample preparation procedures and reducing the problem related to thickness dependence of the resulting spectra [55].

In the present work, the setup employed for this analysis consisted of a FTIR model Nicolet 380 (Thermo Fisher Scientific Italia, Monza, Italy) coupled with a single bounce ZnSe crystal ATR base (MIRacle™ Single Reflection, Pike Technologies). All the analyses were conducted on solid samples, which were directly pressed onto the crystal using calibrated pressure. The spectra were collected by averaging 32 scans with a resolution of 4 cm^−1.^

### 2.4. Water Absorption

Water absorption measurements are of high importance in the case of facilitated transport systems as water actively participates in many of the interactions between the CO_2_ to be transported and the amine-based carriers. This parameter, therefore, often proves useful in understanding the behavior of gas permeation in humid conditions. In the present work, the tests were conducted by using a quartz spring microbalance, which was purposely set up for this type of experiment. The system, described in a previous work, [56] is formed by a quartz spring placed into a water jacketed column for temperature control, connected to a water reservoir and to a vacuum line.

During tests the sample was hung to the spring and treated under vacuum at the experimental temperature to ensure removal of residual water. Once the weight was stabilized, the sample compartment was filled with the desired amount of water vapor, controlled through a pressure transducer, and the absorption was measured by monitoring the weight change of the sample. This was achieved using a CCD camera focused on the sample and connected to a PC thus allowing the continuous recording of the weight changes over time. Once the system reached equilibrium, the pressure was increased stepwise to start a new sorption test. Differential sorption steps continued until a water activity of about 90% was obtained, which was the highest value reachable without incurring in condensation problems. At each step, the equilibrium water uptake, *M_eq_*, could be measured through Equation (1) below:

(1) $$M_{eq}=\left(h_{eq}-h_{0}\right)\cdot \;\frac{k}{g}$$

where *k* is the spring elastic constant, *h_eq_* the spring length at equilibrium (after the absorption step is completed), *h_0_* is the initial spring length and *g* is the gravity acceleration. The buoyancy effect, which could affect the reading, was evaluated but was negligible due to the low pressure considered and for this reason was not introduced in the equation.

The overall precision of the systems was ±5 µg considering the sensitivity of the quartz spring and the resolution of the CCD camera.

Apart from equilibrium sorption, from the mass uptake over time, *M(t)*, the diffusion coefficient *D_w_* of water in the system was also measured by solving Fick’s second law with the condition of constant water concentration on the sample surface. In this condition, the following Equation (2) is valid [57]:

(2) $$\frac{M\left(t\right)}{M_{eq}}=1-\underset{n}{\sum }\frac{8}{{\left(2n+1\right)}^{2}\pi^{2}}exp\left[\frac{-D_{W}{\left(2n+1\right)}^{2}\pi^{2}t}{L^{2}}\right]$$

allowing the calculation of *Dw* once the sample thickness L is known.

### 2.5. Gas Permeation

**Figure 2:**
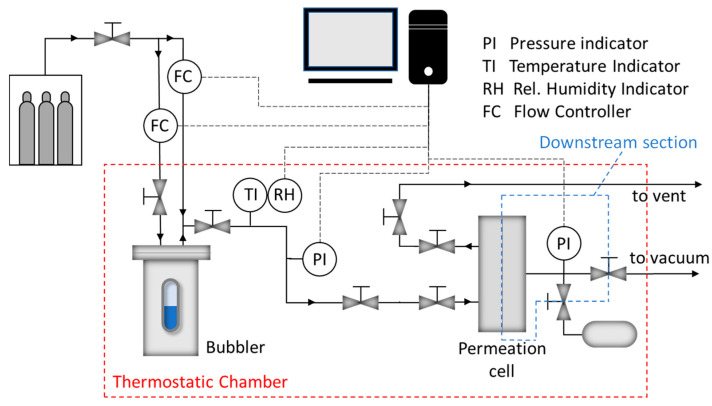
Schematic of the permeation equipment used for testing.

Permeation tests were conducted considering CO_2_, N_2_ and CH_4_ at a temperature of 35 °C and at high humidity, close to 100%; to this end, a permeation apparatus purposely developed to carry out humidified gas experiments was used as described in previous work [58]. The system, schematized in Figure 2, is based on a classical fixed volume variable pressure permeometer but is endowed with a bubbler that can humidify part of the feed thus controlling the water content of the permeating gas. This gas is continuously fed to the cell upstream side to avoid possible polarization concentration phenomena; the downstream volume is closed and calibrated allowing the calculation of the permeate flow through the pressure increase, and concentration polarization problems have, on this side, a negligible effect on the permeation due to the very low pressure usually kept during the experiments.

To ensure that only the gas of interest permeated through the membrane, a humidity equilibration was carried out, setting the water pressure in the upstream and downstream side of the permeation cell to the value corresponding to the humidity to be tested. Using this method, when the experiment was started by feeding the humidified gas on the upstream side of system, the water was in equilibrium at the two sides of the membrane, and its contribution to permeation was substantially negligible.

The permeability (℘) can be therefore calculated with the same equation used in fixed volume variable pressure systems, that is:

(3) $$℘={\left(\frac{dp_{1}}{dt}\right)}_{t\to \infty }\;\frac{V}{RT}\;\frac{L}{A}\;\frac{1}{\left(p_{2}-p_{1}\right)}$$

where *V* is the downstream volume, *L* the membrane’s thickness, *A* the permeation area, *T* the temperature, *p_2_ − p_1_* the pressure gradient between upstream and downstream compartment and *R* the gas constant.

## 3. Results and Discussion

### 3.1. FTIR Analysis

**Figure 3:**
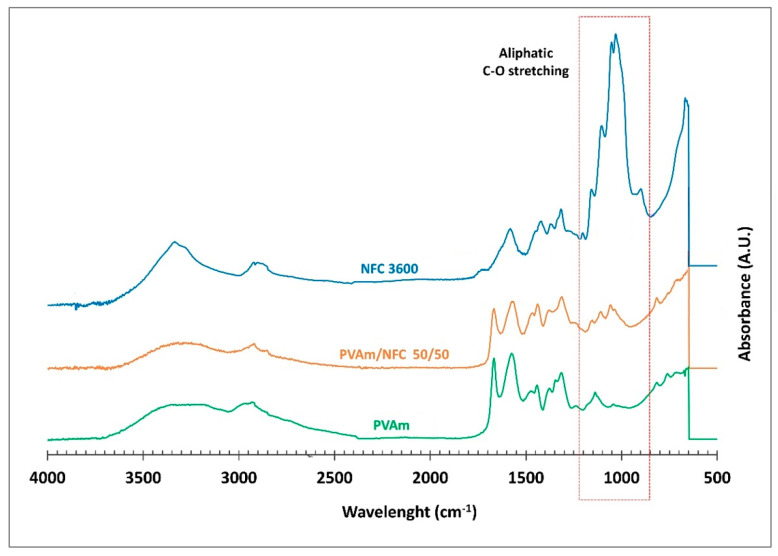
FTIR spectra of cNFC, PVAm and PVAm/cNFC 50/50 blends.

**Figure 4:**
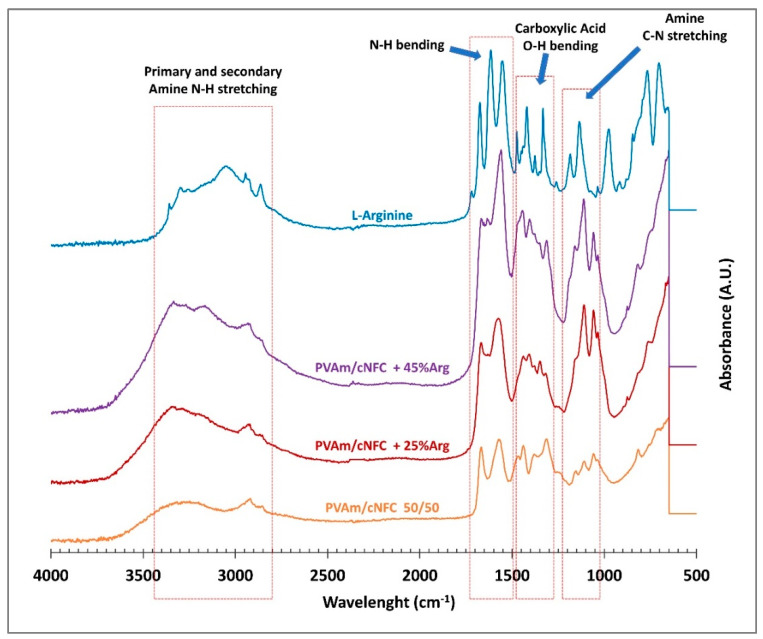
FTIR spectra of ternary PVAm/cNFC+Arg blends, Spectra of binary PVAm/cNFC blend and of L-arginine are also reported for comparison.

The infrared spectra of the different membranes considered in the present work are reported in Figure 3 and Figure 4, together with those of the materials used for blending, that is, cNFC, PVAm and L-arginine.

In particular, in Figure 3, which shows the spectra related to the cNFC-PVAm membrane, it is possible to notice that the 50/50 blend is characterized by all of the characteristic peaks of PVAm such as the 3300 cm^−1^ peak related to the stretching of −NH_2_ groups as well as the N-H bending at 1500–1750 cm^−1^; the fingerprint of the nanocellulose is mainly visible in the region between 800 and 1200 cm^−1^, where no characteristic peaks of PVAm are present, making clear the presence of the peaks at around 1000 cm^−1^, corresponding to the C–O stretching of the cNFC.

The analysis of the spectra may suggest that a low amount of nanocellulose is actually present in the system, as the nanocellulose peaks are smaller with respect to those related to PVAm. However, it should be noticed that due to the material structure, polymer chains in the blends are expected to cover the nanocellulose fibers and fill the surface voids, which are known to exists on nanocellulose films [59]. For this reason, they are expected to exist in a higher concentration on the surface of the films; this is the part mainly sampled by ATR evanescent wave, that is able to penetrate only a very thin layer of the membrane during the test [55].

The FTIR of the cNFC-PVAm-L-arginine blends is reported in Figure 4, where for the sake of comparison the spectra of the 50/50 blends are also shown. In this case, the FTIR spectra are highly affected by the presence of arginine, and the presence of the amino acid is clearly visible in 25 wt% loaded samples: primary and secondary N–H peaks from 2800 to 3400 cm^−1^ are visible, as well as other nitrogen-based signals, like the mentioned N–H bending at 1500–1750 cm^−1^ and C–N stretching at 1100–1200 cm ^−1^. A set of carboxylic acid signals from arginine can be observed as well, between 1500 and 1250 cm – 1, which substantially overlap and cover the nanocellulose peaks. This result suggests that arginine is well dispersed in the PVAm matrix and not only on the fiber surface even if it was added and interacted with the fibers prior to PVAm addition. All the arginine peaks in the spectrum are increased in the sample with 45% loading, confirming the higher concentration of amino acid in this material.

### 3.2. Water Sorption

#### 3.2.1. Solubility Analysis

**Figure 5:**
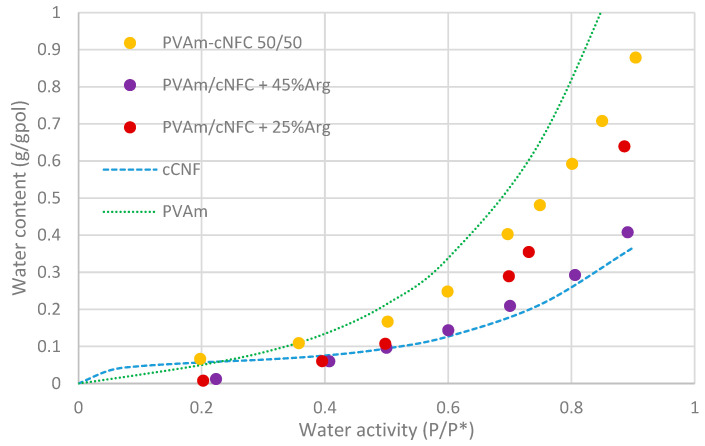
Sorption isotherm of the different systems investigated in the present work. Literature data related to the purified PVAm [25] and investigated nanocellulose [19] are also reported for completeness.

In Figure 5 the water sorption measured at 35 °C in the materials studied in the present work is reported as a function of the water activity (pressure divided by vapor pressure) in the vapor phase. The sorption isotherms referring to nanocellulose and purified Lupamin are also reported for the sake of completeness [19,25].

The data show that, even if the purification process is known to reduce the Lupamin hydrophilicity, [25] PVAm remains the most hydrophilic component among those considered in the present blends; arginine and cNFC are indeed known to have similar water uptake [19]. As a consequence, the different blends investigated generally show lower water uptake with respect to pure PVAm and follow a trend that is strictly related to the PVAm concentration in the matrix. The binary blend PVAm/cNFC with 50% aminated polymer indeed shows higher water solubility with respect to the ternary blends with 25% arginine (and 37.5% PVAm), which in turn has higher uptake with respect to the films with 45% arginine (27.5% PVAm).

The maximum uptake of the three films, measured at about 90% RH, is in the order of 71 wt% for the binary blends and 63% and 41 wt% for the blends containing 25 and 45% arginine, respectively.

Interestingly, the addition of the amino acid not only affected the value of the water uptake but also changed the shape of the isotherm particularly in the low relative humidity range.

At about 20% RH the binary blend shows a rather high water uptake, which suggests the existence of a change in the curvature of the sorption isotherm, similar to the case of pure nanocellulose [41,42], while the ternary blends show lower uptake with a clearly defined upward curvature in the whole activity range inspected.

It is well known that, in pure nanocellulose films, the coexistence of multiple sorption processes creates complex behavior with a downward curvature (Langmuir type) at low RH and an upward curvature (multilayer adsorption or absorption) at high RH [42]. The observed results therefore suggest that the binary blend still presents both type of processes with the second enhanced by PVAm hydrophilicity. The ternary mixtures, on the other hand, lose the uptake related to the adsorption, likely due to a very efficient surface coverage of the active adsorption sites (hydroxyl groups) of nanocellulose fibers by the small amino acid molecules. Therefore, it seems that the interaction between arginine and nanocellulose, on one hand, stabilized the suspension during membrane formation (as mentioned in the preparation section) but, on the other hand, clearly decreased the ability of nanocellulose to bind water. As a consequence, the overall uptake of the ternary blends was reduced, so that these materials showed a lower solubility with respect to cNFC/PVAm blends, especially the films with 45% arginine, which indeed led to water uptake substantially equal to that of pure nanocellulose.

#### 3.2.2. Diffusivity Analysis

**Figure 6:**
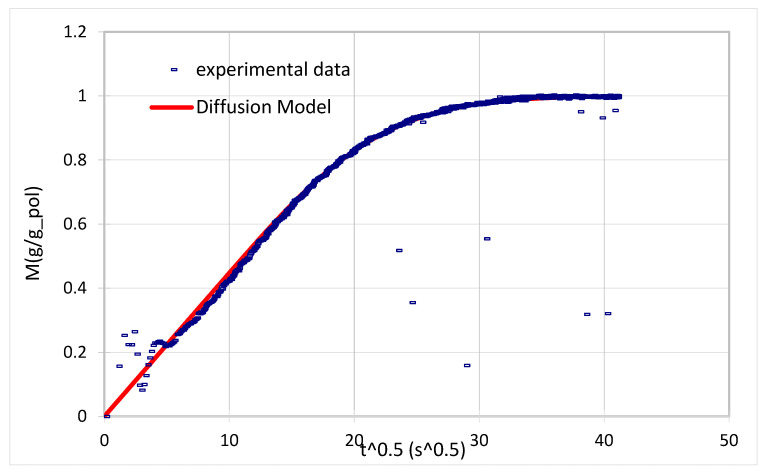
Fitting of the sorption kinetics with the Fickian model (Equation (2)); the data refer to the sorption step between 0.7 and 0.8 activity for the PVAm/cNFC + 45% Arg.

From water sorption tests, information on the diffusion process can be obtained by using Equation (2) to fit data related to the process kinetics. In general, good fitting of the different data and the equations could be obtained, as shown for example in Figure 6 for the PVAm/cNFC + Arg 45% film, confirming the Fickian characteristics of the diffusion process. In particular, the use of purified PVAm seemed to reduce the relaxation tendency of the films, which was clearly observed when Lupamin was used in the blends [33]. Fickian behavior was observed also in the ternary systems, so it can be concluded that the addition of amino acids did not increase the tendency of the films to swell and plasticize. This is in line with the lower water uptake observed for the system containing arginine with respect to the membrane with 50% PVAm.

**Figure 7:**
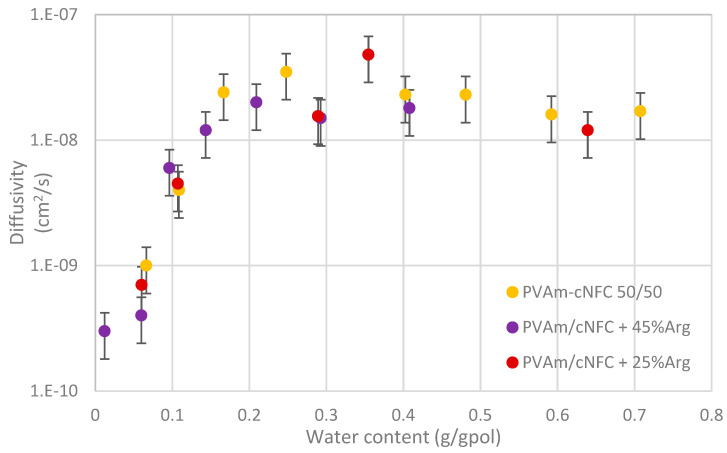
Diffusion coefficient of water in the different inspected materials as obtained through Equation (2).

The general behavior of the diffusion coefficient, as a function of water content, is reported in Figure 7 and remains substantially the same observed for Lupamin–NFC blends and common to other hydrophilic polymers [60,61]. The diffusivity indeed showed an increase in the range of low humidity to then stabilize and remain substantially constant (or slightly decreasing) when the water content was increased.

In particular, values in the order of 5·10^–10^ cm^2^/s up to 5·10^–8^ cm^2^/s, were observed when the humidity was increased from 0 up to 40–50% and water uptake reached 20 wt%, while after this threshold the diffusivity stabilized around the value of 2·10^–8^ cm^2^/s with variations that remained within the uncertainty of the measurements. These values are once again in line with those related to Lupamin–NFC films as well as to arginine–NFC films, suggesting a common transport mechanism for water vapor in all these materials [19,32]. Briefly, the low water diffusivity in the system can be related, at low humidity, to the difficulty of the water molecules to penetrate and break the hydrogen bonding network existing among the different constituents of the films: nanocellulose fibers, aminated polymer chains and amino acid molecules. The progressive weakening of these bonds is therefore accompanied by a continuous increase of the diffusion coefficients, which ends when a continuous pathway of the hydrated polymer is formed within the cellulose fibers. In this condition the diffusive process is no longer hindered by the material structure, and an increase of water uptake does not affect the diffusion coefficient, which actually slightly decreases, likely due to clustering of water molecules within the polymer [61]. Therefore, the water content is expected to affect the gas transport in the materials, not only helping the facilitated transport mechanism but also because of the structural change of the material upon swelling.

### 3.3. Permeation

Following the analysis of the water sorption, the attention was focused on the gas permeability, which is indeed the most interesting characterization for materials developed for possible applications in carbon capture. Therefore, permeation tests were carried out at 35 °C considering CO_2_, N_2_ and CH_4_ in the different materials inspected. Humidity level close to saturation, RH~100%, was considered as this is the condition that is expected to maximize membrane performance.

**Figure 8:**
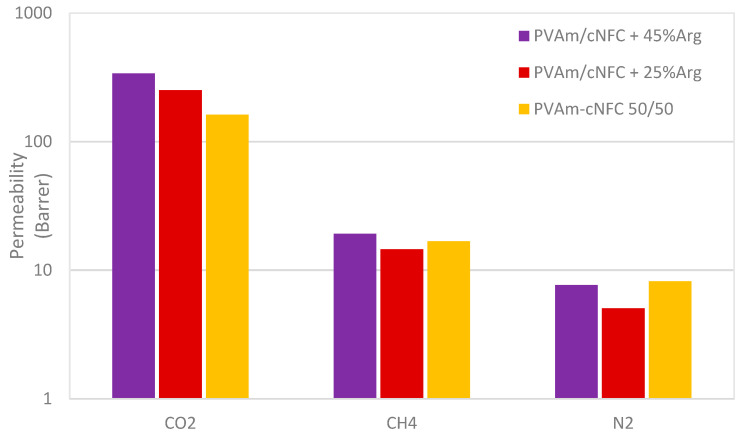
Permeability of methane, nitrogen and carbon dioxide in the different inspected mate-rials as obtained through Equation (3).

The results of the experiments are reported in Figure 8 and show that the addition of the mobile carrier was able to increase both the CO_2_ permeability and selectivity of the PVAm/cNFC membranes with respect to other gases.

Indeed, the CO_2_ permeability increased from about 160 Barrer in PVAm/cNFC binary blends to 250 and 340 Barrer for the materials with 25 and 45% loading of L-arginine. These values are in line with but higher than those observed for cNFC/arginine blends investigated by Venturi et al. [19] who reported a maximum value of 220 Barrer for the films with 45 wt% arginine loading; the positive effect of the PVAm on the material transport properties with respect to CO_2_ is therefore also confirmed in the present case. Interestingly, the observed improvements were obtained despite the decrease of the water content in the ternary membranes with respect to the binary membranes, thus suggesting the improvement was related to the facilitation effect rather than to a plasticization of the membrane due to water content.

This consideration is also confirmed by the analysis of the permeability of other gases, which were only slightly influenced by the presence of the amino acid; the differences among the three materials are, indeed, rather limited with permeability values ranging from 14 to 19 Barrer in the case of methane and from 5 to 8 Barrer in the case of nitrogen.

As a consequence of the different behavior observed for the different gases, the CO_2_ selectivity with respect to methane and nitrogen substantially increases when arginine is added to the membranes from 10 to more than 20 in the case of CO_2_/CH_4_ selectivity and from 20 to about 50 in the case of the CO_2_/N_2_ system.

Unfortunately, these values, while of interest, are lower than those observed in other works (CO_2_/N_2_ selectivity for the cNFC-arginine membranes reported in Reference [19] maxed at 180 at 94% RH), making the present membranes slightly less effective than previous examples in terms of CO_2_ separation potential. This fact seems to be primarily related to the properties of the binary PVAm/cNFC blend that shows selectivity well below that observed for the NFC/PVAm (Lupamin^®^) mixtures with the same composition. Considering data at 90% RH, CO_2_ permeability values in the order of 180 Barrer were obtained with selectivity equal to 50 and 25 for the CO_2_/N_2_ and CO_2_/CH_4_, respectively [33]. The reason for such a difference is still unclear, but it may be related to the use of purified PVAm and highly charged nanocellulose. The strong interaction and the high-speed mixing indeed could cause an almost complete coverage of the NFC fibers by the aminated polymers, thus limiting the formation of the hydrogen bonding network between fibers, which is able to slow down the permeation of inert gases. A more attentive analysis of the influences of such parameters on the overall membrane properties therefore should be conducted in order to further improve the properties of such facilitated transport membranes.

## 4. Conclusions

Facilitated transport membranes obtained by mixing polyvinylamine (PVAm), L-arginine and carboxymethylated nanocellulose (cNFC) were prepared and tested for water sorption and gas permeation. In particular, cNFC/PVAm 50/50 wt% was produced with and without the addition of different amounts of arginine (namely 25 and 45 wt%).

Sorption tests were conducted at 35 °C and up to 90% relative humidity, while permeability was measured for CO_2_, N_2_ and methane at the same temperature and at RH close to 100%.

The sorption results showed that polyvinylamine controls the water uptake, being the most hydrophilic among the different components; the solubility therefore follows the concentration of this polymer in the blend. Water content also affects the water molecule mobility within the matrix as water diffusivity in the different materials shows a very similar behavior when reported as a function of water concentration within the membrane.

The addition of arginine, which acts as a mobile carrier, strongly influenced the CO_2_ permeability in the membranes, which more than doubled (from 160 to more than 300 Barrer) when 45 wt% of this amino acid was added to cNFC/PVAm membranes. Interestingly, the permeability of both nitrogen and methane was only slightly affected by arginine addition so that CO_2_ selectivity also increased substantially from about 20 to 50 for the CO_2_/N_2_ system and from 10 to about 20 in the case of CO_2_/CH_4_.

The observed permeability, with a maximum of about 340 Barrer, is among the highest reported for PVAm/nanocellulose-based membranes, but the observed selectivity is lower than that reported for facilitated transport membranes in general and NFC-based facilitated transport membranes in particular. The latter were reported to have selectivity well above 100, likely due to the different type of nanocellulose used and the different degree of purification of PVam considered. Additional studies are therefore needed to understand the role of these parameters in the membrane behavior in order to optimize further its permselective properties.

## Figures and Tables

**Table 1:** Composition of the membranes considered in the present work (weight %).

Membrane	cCNF	PVAm	Arginine
PVAm/cNFC 50/50	50%	50%	-
PVAM/cNFC + 25% Arg	37.5%	37.5%	25%
PVAM/cNFC + 45% Arg	27.5%	27.5%	45%

## Data Availability

Data is contained within the article.
